# Climbing and Clinging of Urban Lizards are Differentially Affected by Morphology, Temperature, and Substrate

**DOI:** 10.1093/iob/obad006

**Published:** 2023-02-08

**Authors:** P L Vaughn, C Colwell, E H Livingston, W McQueen, C Pettit, S Spears, L Tuhela, E J Gangloff

**Affiliations:** Department of Biological Sciences, Ohio Wesleyan University, Delaware, OH 43015, USA; Department of Biological Sciences, Ohio Wesleyan University, Delaware, OH 43015, USA; Department of Biological Sciences, Ohio Wesleyan University, Delaware, OH 43015, USA; Department of Biological Sciences, Ohio Wesleyan University, Delaware, OH 43015, USA; Department of Biological Sciences, Ohio Wesleyan University, Delaware, OH 43015, USA; Department of Biological Sciences, Ohio Wesleyan University, Delaware, OH 43015, USA; Department of Biological Sciences, Ohio Wesleyan University, Delaware, OH 43015, USA; Department of Biological Sciences, Ohio Wesleyan University, Delaware, OH 43015, USA

## Abstract

Urbanization alters the environment along many dimensions, including changes to structural habitat and thermal regimes. These can present challenges, but may also provide suitable habitat for certain species. Importantly, the functional implications of these habitat shifts can be assessed through the morphology–performance–fitness paradigm, though these relationships are complicated by interactions among habitat choice, other abiotic factors, and morphology across scales (i.e., micromorphology and gross anatomy). The common wall lizard (*Podarcis muralis*) is one example of a cosmopolitan and successful urban colonizer. Quantifying both shifts in morphology over time and morphology–performance relationships under various ecological contexts can provide insight into the success of species in a novel environment. To examine how morphological variation influences performance, we measured seven gross morphological characteristics and utilized scanning electron microscopy to obtain high-resolution images of a claw from individuals living in established populations in Cincinnati, Ohio, USA. We used a geometric morphometric approach to describe variation in claw shape and then compared the claws of contemporary lizards to those of museum specimens collected approximately 40 years ago, finding that claw morphology has not shifted over this time. We then performed laboratory experiments to measure the clinging and climbing performance of lizards on materials that mimic ecologically relevant substrates. Each individual was tested for climbing performance on two substrates (cork and turf) and clinging performance on three substrates (cork, turf, and sandpaper) and at two temperatures (24ºC and 34ºC). Clinging performance was temperature insensitive, but determined by substrate-specific interactions between body dimensions and claw morphology. Conversely, the main determinant of climbing performance was temperature, though lizards with more elongate claws, as described by the primary axis of variation in claw morphology, climbed faster. Additionally, we found strong evidence for within-individual trade-offs between performance measures such that individuals who are better at clinging are worse at climbing and vice versa. These results elucidate the complex interactions shaping organismal performance in different contexts and may provide insight into how certain species are able to colonize novel urban environments.

## Introduction

Urbanization continues to increase around the globe (United Nations, 2018). Urban areas have large and wide-reaching impacts on the organisms that inhabit them, influencing ecological and evolutionary processes (Johnson and Munshi-South 2017; Lambert et al. 2020; Diamond and Martin 2021). This happens through a variety of mechanisms, including via structural habitat modification (Mohan et al. 2011) and changes to properties of the thermal environment (Diamond et al. 2018; Campbell-Staton et al. 2020). Urbanization also causes changes to communities; for instance, urban environments have been shown to enable biological invasions (Ferreira and Tidon 2005; Johnson and Munshi-South 2017; Numbere 2018). Organisms that live in these urban environments can experience selective pressures at an increased strength when compared to natural populations (Doherty et al. 2020; Lambert et al. 2020; Putman and Tippie 2020), increasing the potential for novel evolutionary strategies to emerge. For example, the drastically altered habitat structure in urban environments can exhibit selective pressures (Szulkin et al. 2020) and in turn influence thermal regimes (Campbell-Staton et al. 2020). Such alterations in structural habitat can profoundly impact performance and in turn, morphological evolution (Vanhooydonck and Van Damme 1999; Stuart et al. 2014; Muñoz and Losos 2018; Winchell et al. 2018; Battles et al. 2019; Putman, Gasca, et al. 2019; Putman, Samia, et al. 2019; Donihue et al. 2021).

In lizards, the effects of urban environments on morphology are varied and taxon-specific, including changes in limb length (Winchell et al. 2016; Putman et al. 2019; Gómez-Benitez et al. 2020; Putman and Tippie 2020; Vaughn et al. 2021), body length (Sparkman et al. 2018; Putman, Gasca, et al. 2019), claw dimensions (Falvey et al. 2020; Howell et al. 2022), parasite load and body composition (Lazić 2017), and toe pad dimensions (Winchell et al. 2016). A useful framework to understand causal relationships among morphology, performance, and fitness is the morphology–performance–fitness paradigm, which has been applied in numerous study systems (Arnold 1983; Garland and Losos 1994; Aerts et al. 2000). Past work highlights the importance of placing these relationships in a relevant ecological context—including the role of habitat selection and how morphology–performance relationships can shift in different structural habitat (e.g., Garland and Losos 1994; Calsbeek 2008; Irschick et al. 2008).

Because of its immediate implication for performance in a variety of ecologically important and fitness-relevant tasks, variation in structural habitat can affect the evolutionary trajectory of digits and claws in lizards (Melville and Swain 2000; Tulli et al. 2009, 2011; Yuan et al., 2019, 2020). Arboreal lizards, such as those in the genera *Anolis* and *Gekko*, have evolved frictional adhesive toe pads that allow them to cling to vertical surfaces (Russell 2002; Autumn 2006; Winchell et al. 2018). In environments where *Anolis* lizards moved to higher perches to avoid competition, they rapidly evolved larger toepads and increased lamellae numbers (Stuart et al. 2014; Yuan et al. 2020). These adhesive toepads perform an integral role in allowing these lizards to cling to various surfaces, with larger toepads enabling greater cling force (Donihue et al. 2018; Winchell et al. 2018; Wright et al. 2021). Due to the general trend of urban environments having smoother substrates, anoles and other toepad-bearing lizards who live in those environments tend to develop larger toepads to adhere to these unnaturally smooth surfaces (Winchell et al. 2018; Howell et al. 2022). In addition to toepads, claws play an important role in determining performance through mechanical interlocking of substrate asperities (Cartmill 1974; Dai et al. 2002; Labonte and Federle 2015; Naylor and Higham 2019). To that point, Falvey et al. (2020) found that several species of anoles exhibit similar shifts in claw morphology associated with urban environments. While this provides compelling evidence for the importance of novel habitat structures exhibiting selective pressures on these species, this relationship is relatively understudied in lizards without toepads.

Saxicolous lizards, such as those in the genus *Podarcis*, have evolved short and sharp claws to cling to the rock faces they inhabit (Brown, Gist, et al. 1995; Tulli et al. 2011; D'Amore et al. 2018). The common wall lizard (*Podarcis muralis* Laurenti, 1768) (Squamata: Lacertidae), is a small diurnal lizard endemic throughout much of southern Europe (Speybroeck et al. 2016). An excellent invader, it has successfully established in England (Michaelides et al. 2015), Germany (Heym 2013), and multiple sites in North America, including Cincinnati, Ohio, USA (Hedeen 1984; Brown, Gist, et al. 1995; Davis 2011). *Podarcis muralis* was introduced to urban Cincinnati in the early 1950s, when approximately 10 individuals were released into a yard following a family holiday to Northern Italy (Hedeen 1984; Brown, Gist, et al. 1995; Davis 2011). In the ∼70 years following their introduction, *P. muralis* has firmly established itself in the Cincinnati metro area, with their population exploding to hundreds of thousands of individuals (Davis 2011; Davis et al. 2021). *Podarcis muralis* is considered a climbing specialist and, without the benefit of adhesive toepads, they mainly rely on the interaction of their claws with the large variety of substrates they experience in their environment to climb and produce clinging force (Braña 2003; Druelle et al. 2019).

With lizards from these established populations, we conducted laboratory measurements to quantify the relative influences of body morphology, claw morphology, structural habitat, and temperature on ecologically important performance measures. The ability to both cling and climb is extremely important to *Podarcis*, given their saxicolous lifestyle and preference for vertical surfaces (Brown, Gist, et al. 1995; Van Damme et al. 1997; Zani 2000, Zani 2001; Druelle et al. 2019; Davis et al. 2021; Vaughn et al. 2021). First, by comparing the claw morphology of historical specimens with present-day lizards, we tested the hypothesis that claw morphology has shifted over time, which would suggest selection on claw shape. Previous work demonstrates that such traits are evolutionarily labile both within and across species, suggesting that changes in habitat use could drive intraspecific variation (Tulli et al. 2011; Muñoz and Losos 2018). In support of this idea, our previous work has demonstrated that these lizards have undergone a dramatic shift in their body morphology over time (Vaughn et al. 2021), leading us to believe that claw morphology would change as well. Second, we conducted laboratory performance measurements to quantify the relative influences of body morphology, claw morphology, structural habitat, and temperature on ecologically important performance measures. We predicted the following: (1) Body morphology will be important for climbing performance whereby lizards with longer limbs will climb faster, as previous work in Lacertid lizards demonstrates that morphology favorable for sprinting also benefits climbing (Van Damme et al. 1997). Clinging performance will be determined by a combination of body and claw morphology, whereby individuals with short claws and long limbs display increased clinging performance, in concordance with previous interspecific comparisons (Goodman et al. 2008; Tulli et al. 2011). (2) Due to increased demand for rapid muscle function, climbing will be more temperature-sensitive than clinging, which relies on mechanical interlocking with the substrate. (3) Due to morphological limitations and disparities between performance types, lizards will exhibit within-individual trade-offs such that individuals who display substantial climbing performance will display reduced clinging performance and vice versa. In our previous work, we found various trade-offs between performance conditions (Vaughn et al. 2021), leading us to believe that there would be trade-offs between performance types as well, similar to results in other species (Losos et al. 1993). Taken together, our goal is to highlight trade-offs and the potentially complex interactions across scales that shape morphology–performance relationships as organisms colonize novel environments.

## Methods

### Animal collection and body morphology

From June 7 to July 19, 2021, we collected adult male *P. muralis* (*N* = 29) from six sites within Cincinnati and brought them to the lab at Ohio Wesleyan University for morphology and performance measures (Table S1; as in Vaughn et al. 2021). Utilizing digital calipers (model CD-6, Mitutoyo, Japan) with precision to the nearest 0.01 mm, one experimenter (E.J.G.) measured gross morphological structures following the methods of Vaughn et al. (2021), including snout-vent length (SVL), scapular girdle width (SG), pelvic girdle width (PG), and head length (HL). Additionally, we directly measured the metatarsus, fourth digit, zeugopodium, and stylopodium lengths for all four limbs. These measures were combined into four morphological dimensions to prevent overweighting limb dimensions in further analyses. Our combined measures were front limb length (FrontLimb), front foot length (FrontFoot), hind limb length (HindLimb), and hind foot length (HindFoot). FrontLimb and HindLimb were calculated by taking the average of the left and right anterior/posterior limb lengths (stylopodium length + zeugopodium length). FrontFoot and HindFoot were calculated by taking the average of the left and right anterior/posterior foot lengths (metatarsus length + fourth digit length). Each morphological measurement was taken twice and re-measured if the coefficient of variation was larger than 10%. For subsequent analyses, the average of these two measurements was used. To account for variation in absolute size and allometric scaling of body parts, we created a log_10_–log_10_ regression of each measurement on SVL and used the residual value in all downstream analyses (Kaliontzopoulou et al. 2007; Tulli et al. 2011; Muñoz and Losos 2018). Only individuals with complete sets of toes and claws were included in the experiment.

### Claw imaging

We used scanning electron microscopy (SEM) to create high-resolution images of lizard claws. While this method has been applied to quantify the microscopic morphology of digital structures like toepads (Autumn 2006; Tomasko 2018; Koppetsch et al. 2020; Wright 2020; Garner et al. 2021; Garner et al. 2022), to our knowledge it has not been used to quantify the micromorphology of lizard digits without toepads. The fourth digit on the right rear foot (pes) was collected from live individuals (*N* = 29) and historical specimens (*N* = 12) with dissecting scissors. Digits were collected from live individuals after performance was measured (see below). Interspecific studies have shown that the morphology of this digit and its associated claw are linked to changes in habitat use, namely increased arboreality (Ribas et al. 2004; D'Amore et al. 2018; Falvey et al. 2020; Yuan et al. 2020). Museum specimens were from the collection of the Cincinnati Museum Center, originating from populations in the metropolitan Cincinnati area between 1981 and 1993 (full details in Table S2). After collection, samples were dehydrated through an ethanol dilution series (from 30 to 100% ethanol). Ethanol improves preservation and maintains cell shape of biological samples when compared to methanol and methyl-ethanol (Nikara et al. 2020). Dehydrated samples were prepared for SEM through critical point drying and gold sputter coating to ensure image quality. Samples were placed into microporous specimen capsules while suspended in pure ethanol and loaded into a critical point dryer (Tousimis Samdri-795, Tousimis Research Corporation, Rockville, Maryland, USA). The samples were then positioned laterally and mounted on 13 mm diameter aluminum stubs using carbon-based Leit tabs. Mounted samples were coated in gold for 60 s using the Spi Supplies SPI-MODULE Control and Sputter Coater (Structure Probe, Inc., West Chester, Pennsylvania, USA). Samples were maintained in a desiccator to prevent rehydration and imaged using a ZEISS EVO LS10 SEM (Carl Zeiss Microscopy, LLC, White Plains, New York, USA). Digit scales were left intact and images were obtained with focus on claw shape. Accelerating voltage was maintained between 15 and 20 kV. A *Z* of approximately 29.2 mm was maintained, as well as working distance between 5 and 10 mm. We obtained images at varying magnifications for all samples and included a scale bar in each image.

### Performance measurements

Only lizards that possessed original-growth or fully regenerated tails were considered eligible for experimentation because the tail influences locomotor performance and limb kinematics (Brown Taylor et al. 1995; Jagnandan et al. 2014). Lizards were collected and tested in discrete batches to minimize time in captivity before experimentation. Our previous work (Vaughn et al. 2021) describes the methods of capture and maintenance of the lizard colony. All performance tests were made between 7 and 27 days post-capture. We utilized a vertical racetrack (climbing distance, 1.25 m) outfitted with photocells (Trackmate Racing, Surrey, British Columbia, Canada) at 25-cm intervals and with interchangeable substrates to measure climbing performance. To measure clinging performance, we pulled lizards across a patch of substrate (∼20 cm), using a string harness tied around the pelvis anterior to the rear limbs (Tulli et al. 2011; Schwarz et al. 2021), which permitted lizards to engage all four limbs on the substrate. A single researcher (P.L.V.) used a Pesola Medio-Line Spring Scale (model #40,300, Pesola AG, Switzerland) equipped with a slide marker to record maximum clinging force during a trial. We tested clinging performance on cork, turf, and sandpaper (60 grit; grains ∼400 µm in diameter) and climbing performance on cork and turf (lizards were unable to climb vertically on sandpaper). These substrates generally mimic a range of conditions lizards experience in nature, including bark, short vegetation, asphalt, and rock (pers. obs.). Before each measure, lizards were kept in an incubator for at least 1 h at either cool (24°C) or warm (34°C) temperatures, sufficient time for body temperatures to equilibrate (Zajitschek et al. 2012; Telemeco et al. 2022). We selected these temperatures because they are within the range of active body temperatures of animals in these populations, with 34°C approximating the mean field body temperature for these populations (pers. obs.) and close to the optimal temperature for sprint speed in common wall lizards from populations in France (Telemeco et al. 2022). All performance measures were carried out during active daylight hours (between 10h00 and 16h00) in a climate-controlled room (air temperature mean ± SE: 19.8 ± 0. 4°C). Lizards experienced the performance trials in a random order and each day the order in which each animal underwent trials was randomized. For both clinging and climbing, we measured each lizard three times in quick succession and used the maximum performance value in analysis. For climbing performance, we used the fastest speed over a 25-cm interval, which is within the range of observed field movements (Braña 2003; Monasterio et al. 2009, pers. obs.). We withheld food from lizards for 48 h before each performance measure to ensure a postabsorptive state (Van Damme et al. 1991; Angilletta 2001) and conducted up to two performance measures on an individual each day for up to three consecutive days. All research was conducted under Ohio Division of Wildlife Wild Animal Permit (23-014) and all procedures were approved by Ohio Wesleyan University IACUC (12-2020-02).

### Statistical methods

All statistical analyses were conducted in the R Programming Language (R Core Team 2022). We conducted a PCA with the *prcomp* function to describe the major axes of variation in seven aspects of scaled body morphology. We used a semi-landmark approach to quantify claw shape (Tinius and Russell, 2017; Falvey et al. 2020). A single researcher (P.L.V.) placed semi-landmarks along the claw using TPSDig (Rohlf 2006). Curves were placed along the dorsal and ventral surfaces of the claw using the *draw curves* function. Subsequently, 30 evenly spaced semi-landmarks were distributed along each curve using the *resample curves* function. If needed, semi-landmark points were further adjusted to better fit the claw and resampled in order to distribute them evenly across the new curve. Landmark files were imported into R using the package “geomorph” (Adams et al. 2021). Additional smoothing was performed by implementing a Chaikin's corner-cutting algorithm in the R package “smoothr” (Strimas-Mackey 2018). Thirty equidistant points were subsequently distributed around the new, smoothed curve using the “geomorph” function *digit.curves* and the overlapping tip point for the dorsal and ventral curves was dropped, leaving 59 evenly spaced semi-landmarks. Using the landmarks, we also extracted several univariate measurements: base height, ventral length (length of the ventral arc from base to tip), curvature, and tip angle. Base height was calculated by measuring the distance between the first semi-landmarks of the ventral and dorsal curves. Ventral length was measured by calculating the distance between a semi-landmark and the subsequent semi-landmark (1–2, 2–3, etc.) along the ventral curve of the claw. These segments were added to obtain the length of the ventral arc. Tip angle and curvature were calculated following the methods of Falvey et al. (2020); Zani (2000).

To analyze the two-dimensional profile shape of the lizard claws, we followed the methods of Falvey et al. (2020). Using the R Package “geomorph,” we aligned the semi-landmarks of the claws and removed absolute size from our data set with a generalized Procrustes analysis (GPA) using function *gpagen*. We then performed a PCA of shape variation with the function *gm.prcomp* with minimized bending energy of semilandmarks along the two curves. We utilized Procrustes ANOVA implemented with the function *procD.lm* to quantify differences between historical and contemporary claw size and shape, including log_10_-transformed SVL as a covariate. We tested variation in shape between historical and contemporary specimens using the function *morphol.disparity*.

We utilized mixed linear models to address our motivating questions regarding the relationships among morphology, temperature, and performance and the effect of different substrates on two performance measures. Clinging and climbing performance were analyzed separately. We created models that included categorical fixed effects of substrate type (cork/turf/sandpaper for clinging and cork/turf for climbing) and the temperature treatment of the individual (cool/warm). Each model also included the individual scores from the first axis of variation (PC1) for body morphology and for claw morphology, which explained significant amount of variation (31% and 45%, respectively) in these traits and allows for tractable model structure with biologically-interpretable results. In addition, both models included a covariate of log_10_-transformed SVL. We started with models including a four-way interaction of body morphology PC1, claw morphology PC1, temperature, and substrate, and all lower-order interactions. We utilized a backward-selection procedure, sequentially removing unimportant interactions from the model (*P* > 0.05) one at a time, then re-running models. We present results of a final model that includes our main effects and significant interactions. We included a random intercept for individual to account for covariation of repeated measures made on the same individual and a random intercept for population to account for covariance of lizards from the same population. Climbing speed was log_10_-transformed before analysis to better approximate the assumption of normal distribution of model residuals. We followed this same procedure with models that included linear dimensions of claw morphology, claw length, and claw curvature, rather than PC scores. The results of models with these linear claw dimensions were qualitatively similar to those using PC scores, and so we present only results of the PC score models here (see Table S4 for results of models with linear measures). We visually inspected and statistically tested for normality of the distribution of residuals. In one model (sprint speed model including PC axes for claw shape), transformation of the dependent variable produced marginally skewed residuals (Shapiro-Wilks test *P* = 0.031), but given the general robustness of mixed models to this assumption (Schielzeth et al. 2020), we do not believe this affects our results or interpretation. We implemented models with the lme4 package (Bates et al. 2015), assessed the importance of fixed effects using type III sums of squares with corrected denominator degrees of freedom for *F*-tests (Kenward and Roger 1997), and conducted *post-hoc* comparisons of least-squares means with the emmeans package (Lenth et al. 2018).

We also estimated pairwise correlations of performance measures made under different conditions using the package corrplot (Wei and Simko 2021). We first standardized climbing and clinging data (mean = 0, standard deviation = 1) to compare measures on different scales. We created a correlation matrix of all 45 pairwise combinations of the 10 performance measures (three clinging tests and two climbing tests at each of two temperatures). We further assessed correlation patterns by categorizing correlations between matched performance measures (cling–cling and climb–climb) and unmatched (cling–climb) and tested the significance of these correlations against a null hypothesis of no correlation (zero) with a one-sample *t*-test. Data figures were created with the packages ggplot2 (Wickham 2016), corrplot (Wei and Simko 2021), and plot3D (Soetaert 2021).

## Results

### Body and claw morphology

**Fig. 1:**
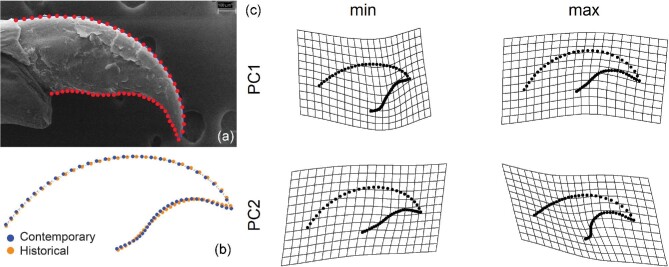
Geometric morphometric analyses of lizard claws (from the fourth digit of right rear foot) in adult male common wall lizards (*Podarcis muralis*). Panel A: placement of semi-landmarks along dorsal and ventral borders of claw on image generated using scanning electron microscopy (SEM). Panel B: consensus shapes from historical (orange) and contemporary (blue) lizards. Panel C: claw shapes with minimum and maximum values on first two principal axes of variation, shown with mesh shapes.

**Fig. 2:**
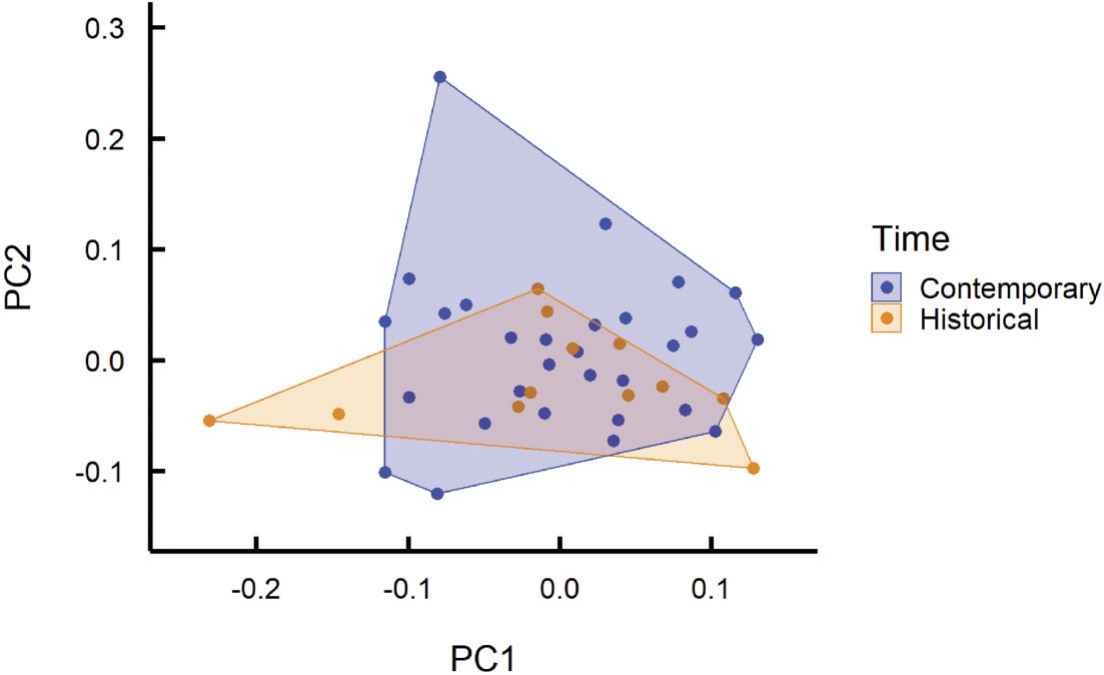
PC plot showing significant overlap on the first two axes of variation in claw morphology for historical (orange) and contemporary (blue) adult male common wall lizards from Cincinnati, Ohio, USA.

The first three axes of variation in body morphology describe 68.9% of total variation. The first axis, describing 30.8% of variation, contrasts lizards with small front limbs and larger feet, pelvic, and shoulder girdles, scoring low on this axis, with lizards having large front limbs and smaller feet, pelvic, and shoulder girdles, scoring high on this axis (Table 1). The second axis of variation, describing 21.6% of variation, contrasts lizards with smaller heads, limbs, and feet and larger pelvic and shoulder girdles, scoring high on this axis, with low-scoring lizards possessing the opposite combination of traits (Table 1). Claw morphology varied with lizard body size, but did not differ between historical and contemporary lizards whether size was included as a covariate or not in the Procrustes ANOVA (log_10_ SVL: *F*_1,40_ = 4.97, *P* = 0.007; Time with SVL as covariate: *F*_1,40_ = 0.82, *P* = 0.46; Time without covariate: *F*_1,40_ = 0.62, *P* = 0.61) nor did we find evidence of shape disparity (differences in within-group variance) between historical and contemporary lizards (*P* = 0.84). Principal component analysis shows that the first two axes of variation explain 76% of the morphological variation, with the first axis describing variation between claws that are short vs. those that are long and the second axis describing variation between claws that are less curved vs. those with that are more curved (Fig. 1). Plotting historical and contemporary lizards onto PC space demonstrates significant overlap between groups (Fig. 2).

**Table 1:** Principal component variable loadings (see text for statistical details)

Proportion of variance explained **Morphological measure**	**PC1** 30.8%	**PC2** 21.6%	**PC3** 16.5%
Pelvic girdle (PG)	−0.435	0.228	−0.276
Shoulder girdle (SG)	−0.501	0.172	0.335
Head length (HL)	−0.025	−0.231	−0.847
Total front limb length (FrontLimb)	0.455	−0.348	0.263
Total front foot length (FrontFoot)	−0.324	−0.574	0.109
Total hind limb length (HindLimb)	0.012	−0.558	−0.002
Total hind foot length (HindFoot)	−0.497	−0.319	0.117

Values shown are first three axes of variation describing the morphological phenotype of adult male common wall lizards (*Podarcis muralis*).

### Performance

**Fig. 3:**
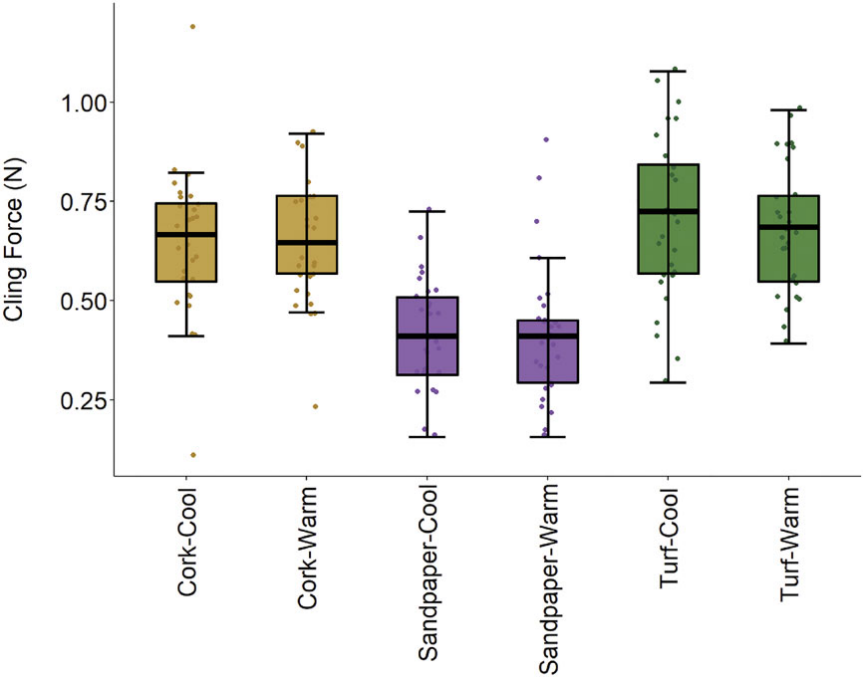
Boxplots and raw values of clinging performance across all substrate and temperature combinations in adult male common wall lizards (*Podarcis muralis*). Lizard clinging performance was insensitive to temperature, while lizards were able to cling with more force on cork and turf compared to sandpaper (see text for details). Tukey boxplots show median, interquartile range, and limits of values within 1.5 times the interquartile range of raw data.

**Fig. 4:**
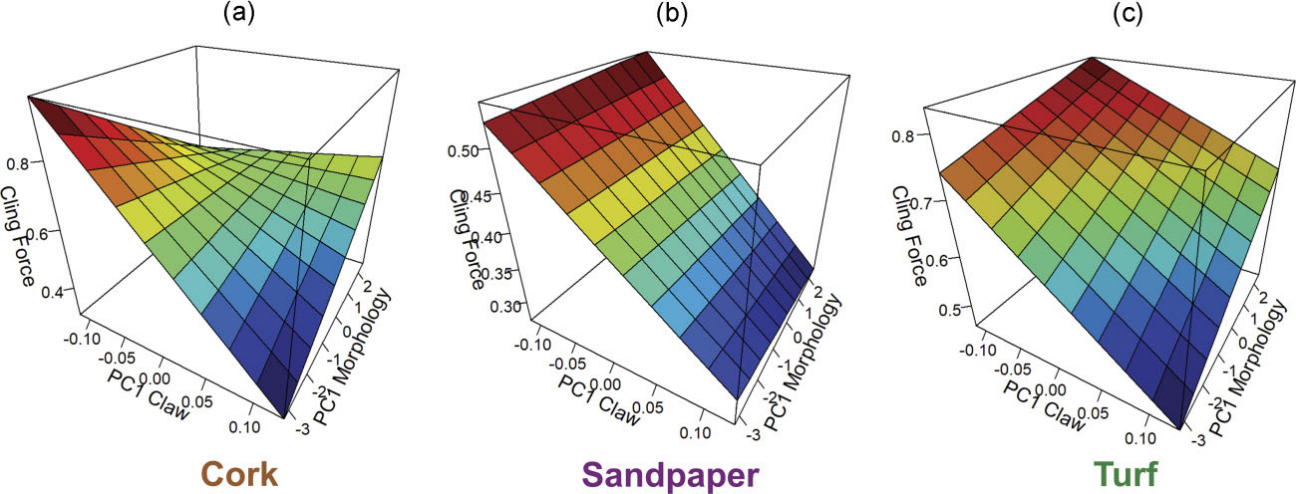
Three-dimensional surface plots demonstrating how the interaction of the first axis of variation in body morphology (*x*-axis) and the first axis of variation in claw morphology (*y*-axis) affect clinging performance (*z*-axis) in adult male common wall lizards (*Podarcis muralis*) on (a) cork, (b) sandpaper, and (c) turf substrates. PC 1 for claw shape represents a continuum of short (low scores) vs. long (high scores) claws. PC 1 for morphology contrasts individuals with large shoulder and pelvic girdles, large feet, and small limbs (low scores) with individuals with small shoulder and pelvic girdles, small feet, and long limbs (high scores).

Clinging performance was influenced by complex interactions between body morphology, claw shape, and substrate (Table 2). Clinging performance was temperature-independent, larger lizards were able to cling with more absolute force, and lizards were able to cling significantly better to cork and turf than to sandpaper (*post-hoc* least-squares means comparisons: cork-sandpaper, *P* < 0.001; turf-sandpaper, *P* < 0.001; and cork-turf, *P* = 0.08; Fig. 3, Table S3). Body morphology interacted with the first axis of claw shape variation (describing longer vs. shorter claws; Fig. 1), but the form of this interaction depended on substrate, with different trait combinations providing maximum performance on different substrates (Fig. 4). Generally, lizards with longer claws performed poorly, but maximum performance was achieved with different body morphologies on cork and turf. Individuals who possessed short claws and high PC1 scores for body morphology produced maximum clinging performance on turf. Individuals who possessed short claws and low PC1 scores for body morphology achieved maximum performance on the cork substrate. Clinging performance on sandpaper was insensitive to body morphology, but shorter claws produced maximum performance. Analysis with linear dimensions of claw curvature and shape rather than principal component axes reveals qualitatively similar patterns (Table S4).

**Table 2:** Results of linear mixed model analyses describing the effect of substrate, temperature, body morphology, multivariate claw morphology, and their interactions on clinging and climbing performance in adult male common wall lizards (*Podarcis muralis*).

Source of variation	Clinging performance	Climbing performance
Substrate		
*F* (*df_n_, df_d_*)	75.6 (2,136)	5.98 (1, 84)
Pr > *F*	< 0.001***	0.017*
Temperature		
*F* (*df_n_, df_d_*)	0.12 (1,136)	104.3 (1, 84)
Pr > *F*	0.723	< 0.001***
Body morphology PC1		
*F* (*df_n_, df_d_*)	0.30 (1, 21)	0.45 (1, 24.5)
Pr > *F*	0.590	0.507
Claw morphology PC1		
*F* (*df_n_, df_d_*)	2.5 (1, 16.4)	6.8 (1, 24.4)
Pr > *F*	0.063	0.015*
SVL *(log_10_)*		
*F (df_n_, df_d_)*	25.9 (1, 23.4)	NA
Pr > *F*	< 0.001***	
Substrate × body morphology PC1		
*F* (*df_n_, df_d_*)	1.9 (2,136)	NA
Pr > *F*	0.160	
Substrate × claw morphology PC1		
*F* (*df_n_, df_d_*)	0.12 (2,136)	4.0 (1, 84)
Pr > *F*	0.885	0.049*
Body morphology PC1 × claw morphology PC1		
*F* (*df_n_, df_d_*)	0.98 (1, 24)	NA
Pr > *F*	0.332	
Substrate x body morphology PC1 × claw morphology PC1		
*F* (*df_n_, df_d_*)	3.2 (2,136)	NA
Pr > *F*	0.043*	

Results show test statistics for fixed effects in final, simplified model (NA indicates effects not retained in final model). Models also included random intercepts to account for repeated measures on individuals and covariance of lizards from the same population. See text for statistical details. Significant effects are designated in bold with one (P < 0.05) or three (P < 0.001) asterisks.

**Fig. 5:**
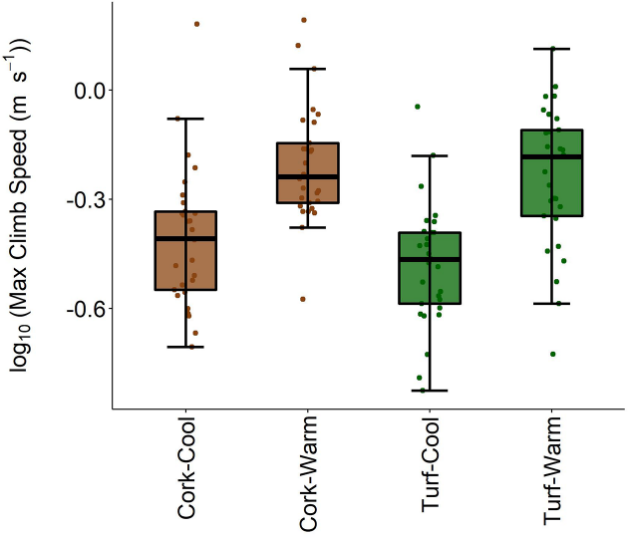
Boxplots and log10-transformed values of climbing performance on a 25-cm interval across all substrate and temperature combinations in adult male common wall lizards (*Podarcis muralis*). Lizards were able to climb faster when warm, while claw shape interacted with substrate to affect climbing performance (see text for details). Tukey boxplots show median, interquartile range, and limits of values within 1.5 times the interquartile range of raw data.

Substrate, temperature, and the interaction of claw shape and substrate were important in determining climbing performance (Table 2). Warmer lizards climbed faster (Fig. 5, Table S3) and lizards with higher scores on the first axis of claw variation (describing lizards with short vs. long claws) were able to climb faster and this effect was stronger on turf (Fig. S1). Neither body size nor body morphology affected climbing performance. Analysis with linear dimensions of claw curvature and shape rather than principal component axes reveals qualitatively similar patterns (Table S4).

**Fig. 6:**
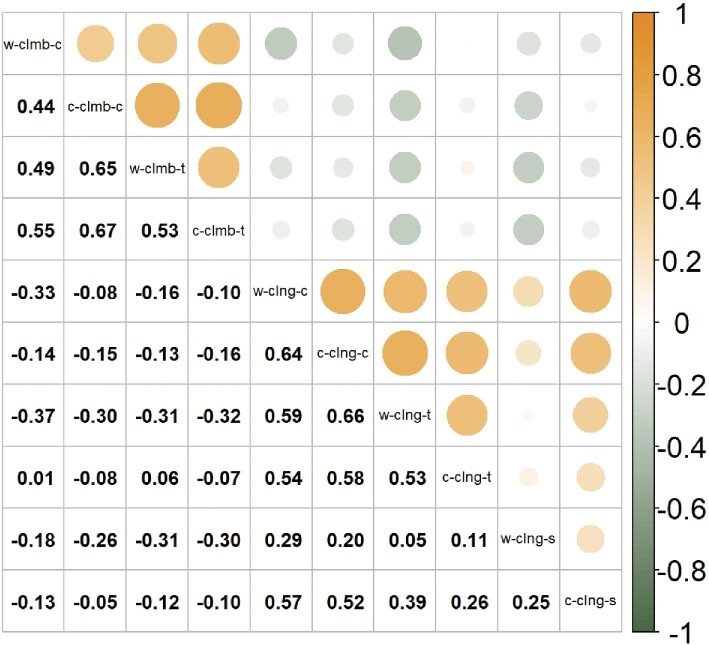
Pairwise correlation matrix of performance measures of adult male common wall lizards (*Podarcis muralis*) between all condition combinations. Above the diagonal shows graphic representation of the strength and direction of correlations (correlations where *P* > 0.05 not shown). Diagonally below diagonal shows correlation estimate. Abbreviated conditions are ordered to indicate temperature (w = warm or c = cool), performance measure (clng = cling, clmb = climb), and substate (c = cork, t = turf, s = sandpaper).

Correlation coefficients for measures of clinging and climbing performance on different substrates and at different temperatures ranged from −0.32 to 0.67 (Fig. 6). Subsetting the correlations by performance measures reveals important patterns in among-individual variation. Correlations between matched performance measures were strongly positively correlated (cling–cling correlations, mean ± SD: 0.41 ± 0.20, range: 0.05–0.66, one-sample *t*-test: *t*_14_ = 7.9, *P* < 0.0001; climb–climb correlations, mean ± SD: 0.56 ± 0.09, range: 0.44–0.67, one-sample *t*-test: *t*_5_ = 15.4, *P* < 0.0001), while correlations between different measures of performance were negatively correlated (cling–climb correlations, mean ± SD: −0.17 ± 0.12, range: −0.37–0.06, one-sample *t*-test: *t*_23_ = −7.12, *P* < 0.0001). Furthermore, correlations of performance measures at different temperatures were positive, providing no evidence of a trade-off in lizard performance at warm vs. cool temperatures (warm–cool correlations, mean ± SD: 0.17 ± 0.35, range: −0.12–0.66, One-sample *t*-test: *t*_24_ = 2.44, *P* = 0.023).

## Discussion

Our experiment demonstrates important morphology-performance associations in a successful urban colonizer, providing insight into the constraints and challenges species will continue to face as urban areas rapidly expand. Our analyses of performance reveals that body temperature is the main determinant of climbing performance whereby lizards with higher body temperatures climb faster. Individuals with longer claws, as indicated by higher PC1 scores for claw morphology, also climb faster. On the other hand, clinging performance is temperature insensitive, but is influenced by both claw and body morphology. Lizards with shorter claws and lower PC1 scores for body morphology (smaller front limbs, longer feet and wider shoulder, and pelvic girdles) displayed the greatest clinging performance on cork, while lizards possessing short claws and higher PC1 scores for body morphology (longer front limbs, shorter feet, narrower girdles) displayed the greatest clinging performance on turf. Surprisingly, individuals with shorter claws had increased clinging performance on sandpaper, but this was insensitive to variation in body morphology (Fig. 4). Claw morphology has not changed over the past ∼40 years, but previous results demonstrate that body morphology has shifted dramatically in these populations (Vaughn et al. 2021). Additionally, we found evidence to support performance trade-offs between the two performance types, suggesting that individuals who perform well at climbing will display decreased clinging performance and vice versa. Given the opposite effects of claw morphology on these two performance measures, claw shape is likely an important mediator of the trade-off between climbing and clinging, which could have important ecological ramifications in both different habitat types and during different activities. Taken together, these results reveal the complexity and context-dependency that underlies morphology–performance relationships.

*Podarcis muralis* is considered a climbing specialist (Druelle et al. 2019) and uses mainly anthropogenic stone structures in urban environments in Cincinnati (unpublished data). Our experimental result is in line with interspecific comparisons among lizard species, which found that arboreal taxa possess longer, taller, and more curved claws than related terrestrial taxa (Ribas et al. 2004; Tulli et al. 2009; Crandell et al. 2014; D'Amore et al. 2018). Paired with the fact that claw shape has not changed over the past four decades, this could be evident to suggest that there has not been a strong selection for claw shape in that period of time. This could be possibly due to the fact that the microenvironment that these lizards are living in has not dramatically changed from their native range to their invasive range in Cincinnati. In urban environments, *P. muralis* utilize anthropogenic stone structures (Brown 1995a; Davis 2011; Druelle et al. 2019), which, especially when sourced from local rocks, can be functionally similar to natural rock outcrops. Pillai et al. (2020) found that coarse (40-grit) sandpaper had a similar surface topography to that of the rocks inhabited by a species of saxicolous gecko. Given that rock surfaces are heterogenous, it is likely that our use of 60-grit sandpaper shares similar topographical characteristics to rocks that *P. muralis* experience in their urban environment. Additionally, the cork and turf substrates are more compliant than sandpaper, allowing lizards to sink their claws in and achieve higher clinging and climbing performance. Due to our limited information on the founding population's source in northern Italy, we do not know how urbanized the original habitat was. The source population could have already adapted to these urbanized substrates in their original environment, leading to a lack of morphological change in the claws in the introduced environment. Another

possibility is that we could be blind to the selection event. Since the introduction event occurred in the 1950s and our historical specimens were collected in the 1980s, the population could have been under strong selection in the ∼30 years before our historical specimens were collected. On the other hand, our previous work demonstrates dramatic shifts in body morphology over this time period, whereby relative HL, pelvic girdle width, hind foot length and front limb length decreased, but relative scapular girdle length increased (Vaughn et al. 2021). The present study demonstrates the importance of variation in limb and girdle dimensions in clinging performance, specifically how body dimensions interact with claw shape differently on different substrates (Table 2, Fig. 4). Lizards with longer front limbs, as described by higher scores on the first axis of variation for body morphology, exhibited better clinging performance on cork and turf. This result is concordant with interspecific studies of clinging performance. For example, arboreal and saxicolous Liolaemini lizards with longer forelimbs exhibit higher clinging force (Tulli et al. 2011) and skinks with longer limbs occupy rock habitats and cling better compared to related species that occupy different habitats (Goodman et al. 2008). Our work indicates that intraspecific morphological variation at different scales (claw vs. body) may interact to shape performance in a context-dependent manner, rendering challenging the identification of clear patterns of selective pressures on these traits in relation to performance measures.

Temperature is key in determining the performance capabilities of ectotherms, but this relationship varies among performance measures (Angilletta et al. 2002; Irschick et al. 2006). Our results show that while climbing performance is temperature-dependent, clinging performance is temperature-insensitive. This could be due to differences in the mechanistic basis of these performance measures. Climbing ability is an active process, relying on muscle output to generate upward force. On the other hand, clinging performance is a more passive process, relying on the mechanical interactions between claws and substrate. This relationship between the claws and substrate could provide insight into why *P. muralis* performs suboptimally on the sandpaper substrate. The small, granular particles on the sandpaper could be so small that the claws cannot get any purchase, essentially leading to an extremely rough substrate becoming functionally smooth (Stark and Yanoviak 2020). Increase in global temperatures can be amplified by urban heat islands, which exert especially strong impacts on urban fauna (Chick et al. 2019; Campbell-Staton et al. 2020; Hall and Warner, 2018, 2019; Rizwan et al. 2008). Our results show that rising temperatures will likely cause trait-dependent shifts in morphology–performance relationships and, depending on the ecological niches of the organisms, could impact long-term trait changes and the viability of populations affected (e.g., Muñoz and Losos 2018).

Taken together, our results demonstrate that morphology–performance relationships are shaped by complex interactions among a variety of factors, including temperature and structural environment (Garland and Losos 1994). This points to the need for further research into identifying these interacting variables and examining their functional consequences in different ecological contexts. Further experimentation should be directed at identifying to what degree other performance measures display temperature sensitivity and measuring performance over a larger variety of temperatures to create thermal performance curves for each performance aspect (Lailvaux and Irschick 2007; Lailvaux and Husak 2014). Additionally, further work should be done to quantify the functional implications of morphological responses to urban environments (Winchell et al. 2016; Winchell et al. 2018; Falvey et al. 2020; Avilés-Rodríguez et al. 2021) and how these patterns might differ across taxa.

## Supplementary Material

obad006_Supplemental_Files

## Data Availability

The datasets and R-code supporting this article have been made available through Mendeley Data archive (https://doi.org/10.17632/bf58fznfx2.1).
