# Supplementary material for: Climbing and Clinging of Urban Lizards are Differentially Affected by Morphology, Temperature, and Substrate
Source: Integr Org Biol. 2023 Feb 8;5(1):obad006. doi: 10.1093/iob/obad006 (PMC9952060; doi:10.1093/iob/obad006)
Supplement: obad006_Supplemental_Files [file obad006_supplemental_files.zip › Figure_Captions.docx]

**Fig. 1.** Geometric morphometric analyses of lizard claws (from 4^th^ digit of right rear foot) in adult male common wall lizards (*Podarcis muralis*). Panel A: Placement of semi-landmarks along dorsal and ventral borders of claw on image generated using scanning electron microscopy (SEM). Panel B: Consensus shapes from historical (orange) and contemporary (blue) lizards. Panel C: Claw shapes with minimum and maximum values on first two principal axes of variation, shown with mesh shapes.

**Fig. 2.** PC plot showing significant overlap on the first two axes of variation in claw morphology for historical (orange) and contemporary (blue) adult male common wall lizards from Cincinnati, Ohio, USA.

**Fig. 3.** Boxplots and raw values of clinging performance across all substrate and temperature combinations in adult male common wall lizards (*Podarcis muralis*). Lizard clinging performance was insensitive to temperature, while lizards were able to cling with more force on cork and turf compared to sandpaper (see text for details). Tukey boxplots show median, interquartile range, and limits of values within 1.5 times the interquartile range of raw data.

**Fig. 4.** Three-dimensional surface plots demonstrating how the interaction of the first axis of variation in body morphology (x-axis) and the first axis of variation in claw morphology (y-axis) affect clinging performance (z-axis) in adult male common wall lizards (*Podarcis muralis*) on (A) cork, (B) sandpaper, and (C) turf substrates. PC 1 for claw shape represents a continuum of short (low scores) vs. long (high scores) claws. PC 1 for morphology contrasts individuals with large shoulder and pelvic girdles, large feet, and small limbs (low scores) with individuals with small shoulder and pelvic girdles, small feet, and long limbs (high scores).

**Fig. 5.** Boxplots and log10-transformed values of climbing performance on a 25-cm interval across all substrate and temperature combinations in adult male common wall lizards (*Podarcis muralis*). Lizards were able to climb faster when warm, while claw shape interacted with substrate to affect climbing performance (see text for details). Tukey boxplots show median, interquartile range, and limits of values within 1.5 times the interquartile range of raw data.

**Fig. 6.** Pairwise correlation matrix of performance measures of adult male common wall lizards (*Podarcis muralis*) between all condition combinations. Above the diagonal shows graphic representation of the strength and direction of correlations (correlations where P > 0.05 not shown). Below diagonal shows correlation estimate. Abbreviated conditions are ordered to indicate temperature (h = warm or c = cool), performance measure (clng = cling, clmb = climb), and substate (c = cork, t = turf, s = sandpaper).
