# Supplementary material for: Climbing and Clinging of Urban Lizards are Differentially Affected by Morphology, Temperature, and Substrate
Source: Integr Org Biol. 2023 Feb 8;5(1):obad006. doi: 10.1093/iob/obad006 (PMC9952060; doi:10.1093/iob/obad006)
Supplement: obad006_Supplemental_Files [file obad006_supplemental_files.zip › Vaughn_ClingClimb_IOB_Supplemental_(FINAL).docx]

**Supporting Information For:**

*Climbing and clinging of urban lizards are differentially affected by morphology, temperature, and substrate*

Published in ***Integrative Organismal Biology***, 2023

Princeton L. Vaughn, Caitlyn Colwell, Ethan H. Livingston, Wyatt McQueen, Ciara Pettit, Sierra Spears, Laura Tuhela, Eric J. Gangloff

**Table S1.** Habitat and sample size information from sampled contemporary populations of *Podarcis muralis* in Cincinnati, Ohio, USA.

| Name | Latitude  Longitude | Habitat Type | *N* |
| --- | --- | --- | --- |
| Alms Park | 39°06'41.1"N 84°25'49.3"W | Stone Stairs, Stone Wall, Concrete Block | 2 |
| Ault Park | 39°08'06.7"N  84°24'24.1"W | Stone Stairs, Stone Wall, Concrete Block | 8 |
| Madisonville | 39°09'26.0"N  84°23'37.1"W | Stone Wall, Soil Slope | 7 |
| Mistletoe | 39°06'43.8"N  84°33'05.0"W | Stone Wall | 2 |
| Mount Echo Park | 39°05'35.9"N  84°33'52.2"W | Sidewalks, Concrete walls, Stone buildings, Ornamental vegetation | 4 |
| River Road | 39°05'58.1"N  84°33'20.6"W | Stone Wall | 6 |

**Table S2.**  Historical specimen *Podarcis muralis* ID numbers, GPS coordinates, location name, and year as assigned by the Cincinnati Museum Center. GPS coordinates approximated from collection notes.

| ID | Latitude  Longitude | Location Name | Collection Date |
| --- | --- | --- | --- |
| 3263 | 39°06'58.9"N  84°25'03.8"W | Eastern Ave | 17-Jul-1981 |
| 3271 | 39°06'42.6"N  84°26'06.3"W | 3804 Eastern Ave | 5-Aug-1981 |
| 3286 | 39°06'42.6"N  84°26'06.3"W | 3804 Eastern Ave | 29-Jun-1981 |
| 3295 | 39°06'42.6"N  84°26'06.3"W | 3804 Eastern Ave | 19-Apr-1982 |
| 3307 | 39°06'42.6"N  84°26'06.3"W | 3804 Eastern Ave | 20-May-1983 |
| 3309 | 39°06'42.6"N  84°26'06.3"W | 3804 Eastern Ave | 18-Jun-1983 |
| 3310 | 39°06'42.6"N  84°26'06.3"W | 3804 Eastern Ave | 18-Jun-1983 |
| 3587 | 39°07'22.5"N 84°27'43.3"W | Shore of Ohio Reiver Below Lumbar St | 12-Apr-1987 |
| 3592 | 39°07'18.9"N  84°28'20.9"W | Shore of Ohio River between Lumbar Street and Gotham Place | 25-Apr-1987 |
| 3593 | 39°07'21.6"N 84°27'36.4"W | Shore of Ohio River between Lumbar Street and Gotham Place | 14-May-1987 |
| 3596 | 39°07'21.6"N 84°27'36.4"W | Shore of Ohio River between Lumbar Street and Gotham Place | 4-Sep-1987 |
| 3897 | 39°05'35.0"N  84°26'57.1"W | 234 Clover Ridge Ave | 22-Sep-1993 |

**Table S3.** Least-squares means estimated from mixed linear models of clinging performance (on cork, sandpaper, and turf) and climbing performance (on cork and turf) in adult male common wall lizards (*Podarcis muralis*). See text for statistical details.

| Substrate | Temperature | Clinging Force  Least-squares mean (confidence limits) | Climbing Speed (log_10_-transformed)  Least-squares mean (confidence limits) |
| --- | --- | --- | --- |
| cork | cool | 0.650 (0.603 — 0.697) | -0.396 (-0.510 — -0.283) |
| sandpaper | cool | 0.425 (0.378 — 0.472) | NA |
| turf | cool | 0.702 (0.654 — 0.749) | -0.448 (-0.562 — -0.335) |
| cork | warm | 0.643 (0.596 — 0.690) | -0.174 (-0.288 — -0.061) |
| sandpaper | warm | 0.418 (0.371 — 0.465) | NA |
| turf | warm | 0.695 (0.648 — 0.742) | -0.226 (-0.340 — -0.113) |

**Table S4.** Results of linear mixed model analysis describing the effect of substrate, temperature, body morphology, and univariate claw morphology and their interactions on clinging and climbing performance in adult male common wall lizards (*Podarcis muralis*). Model also included a random intercept of individual to account for repeated measures. Results shown show test statistics for fixed effects in final, simplified model (NA indicates effects not retained in final model). Models also included random intercepts to account for repeated measures on individuals and covariance of lizards from the same population. See text for statistical details.

| **Source of Variation** |  |  | **Clinging Performance** | | **Climbing Performance** |
| --- | --- | --- | --- | --- | --- |
| ***Substrate*** | |  | |  |  |
| *F* (*df_n_, df_d_*) |  |  | 74.0 (2, 142) | | 5.5 (1, 85) |
| Pr > F |  |  | **<0.001***** | | **0.021*** |
|  | |  | |  |  |
| ***Temperature*** | |  | |  |  |
| *F* (*df_n_, df_d_*) |  |  | 0.12 (1, 142) | | 100.7 (1, 85) |
| Pr > F |  |  | 0.735 | | **<0.001***** |
|  | |  | |  |  |
| ***Body Morphology PC1*** | |  | |  |  |
| *F* (*df_n_, df_d_*) |  |  | 0.15 (1, 22.2) | | 0.29 (1, 23.9) |
| Pr > F |  |  | 0.703 | | 0.593 |
|  | |  | |  |  |
| ***Claw Ventral Curvature*** | |  | |  |  |
| *F* (*df_n_, df_d_*) |  |  | 0.23 (1, 20.2) | | 0.13 (1, 24.0) |
| Pr > F |  |  | 0.635 | | 0.720 |
|  | |  | |  |  |
| ***Residual Claw Length*** | |  | |  |  |
| *F* (*df_n_, df_d_*) |  |  | 0.05 (1, 24) | | 0.27 (1, 22.3) |
| Pr > F |  |  | 0.824 | | 0.606 |
|  | |  | |  |  |
| ***SVL (log_10_)*** | |  | |  |  |
| *F* (*df_n_, df_d_*) |  |  | 31.2 (1, 22.5) | | NA |
| Pr > F |  |  | **< 0.001***** | |  |
|  |  |  |  | |  |


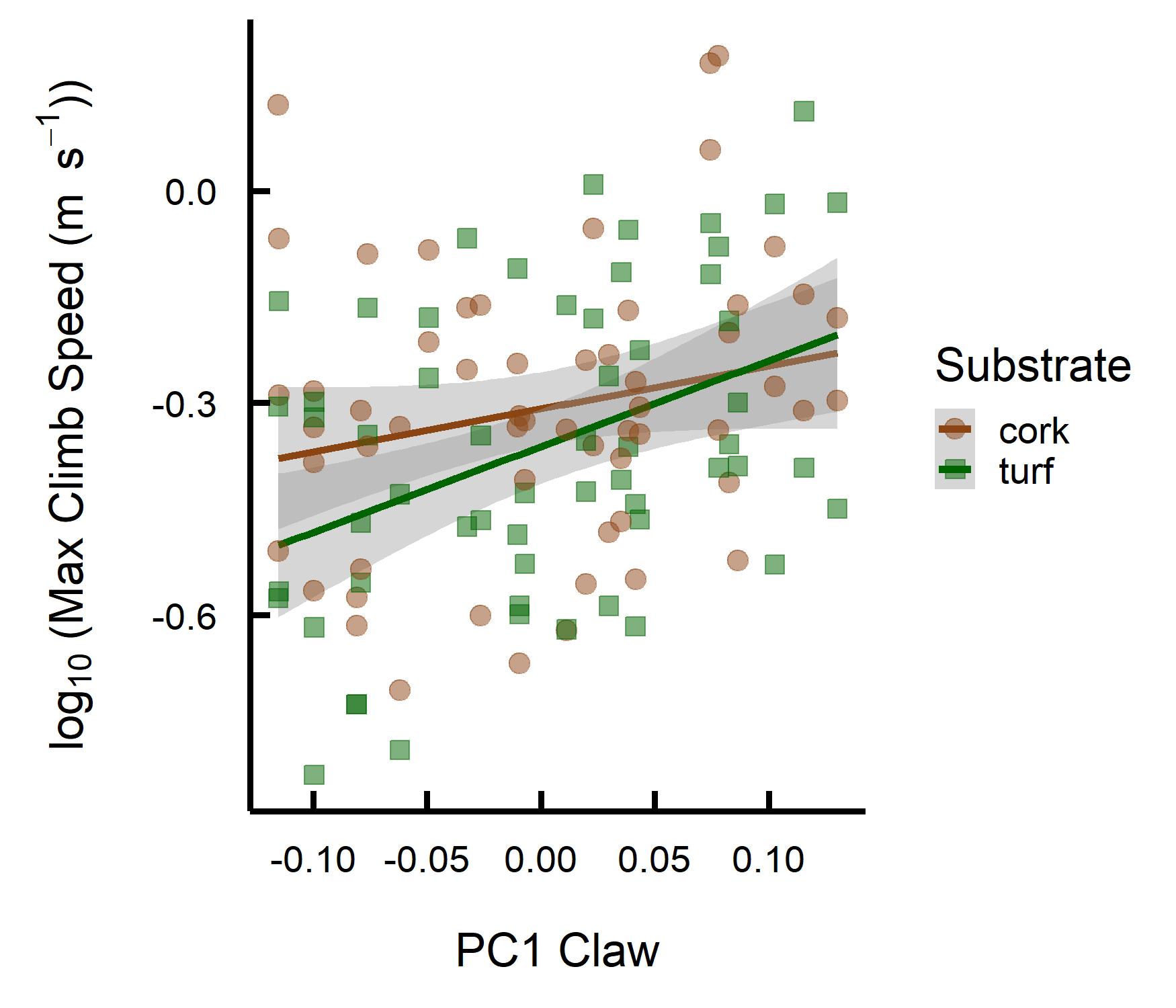


**Fig. S1.** Climbing performance of adult male common wall lizards (*Podarcis muralis*) as a function of PC1 Claw scores (see text for statistical details). Climbing speed combines data across both warm and cool body temperatures for both substrate types. Regression lines shown to demonstrate significant PC1 Claw × Substrate interaction.
